# 154 Avoidance of Physical Activity Among Patients with Chronic Obstructive Pulmonary Disease (COPD): An Observational Study

**DOI:** 10.1093/eurpub/ckae114.150

**Published:** 2024-09-26

**Authors:** Dea Kejlberg Andelius, Anders Løkke, Julie Sandell Jacobsen, Rasmus Østergaard Nielsen

**Affiliations:** Research Unit of General Practice, Aarhus, Denmark; Aarhus University, Aarhus, Denmark; Little Belt Hospital, Vejle; University of Southern Denmark, Odense; Research Unit of General Practice, Aarhus, Denmark; VIA University College, Aarhus, Denmark; Research Unit of General Practice, Aarhus, Denmark; Aarhus University, Aarhus, Denmark

## Abstract

**Purpose:**

While physical activity is recognized as an essential component in COPD treatment, patients with COPD often exhibit lower levels of physical activity compared with their lung-healthy peers. The primary objective of our study was to describe the proportion of patients with COPD who report avoidance behavior related to physical activity.

**Methods:**

A questionnaire-based, observational study was conducted among patients with COPD, scheduled to attend a pulmonary rehabilitation program. Participants completed the COPD Anxiety Questionnaire (CAF-R) and functional tests were conducted prior to participation of the rehabilitation program.

**Results:**

We included 48 participants (24 males, mean: age 70±7.8 years, body mass index 25±5.0, MRC dyspnoea scale 2.5±0.9, One-Minute-Sit-To-Stand-Test 19 ±6.0 repetitions). Findings revealed that 60% (95% CI [46%-0,74%]) reported avoidance of physical exertion, 44% (95% CI [24%-52%]) avoid activities causing breathlessness, and 35% (95% CI [22%-48%]) try avoid any form of physical activity.

**Conclusions:**

Our findings show that a large proportion of patients with COPD exhibit avoidance behavior concerning physical activity. While the underlying reasons are likely multifaceted, apprehension towards breathlessness emerges as a potential contributing factor. The results underscore the importance of investigating avoidance behavior in people with COPD to better understand how to promote physical activity within this population. Future interventions designed to increase physical activity in patients with COPD should include identification of the extent of avoidance behavior and implementation of strategies to manage the behavior, such as exposing individuals to physical activity within a secure environment.

**Support/funding:**

This study received financial support from the Karen Elise Jensen Foundation and the Eva Merethe Falck Crones Foundation.

